# Anticipated Imitation Is Not Affected by the Number of Imitators

**DOI:** 10.1027/1618-3169/a000637

**Published:** 2025-04-01

**Authors:** Bence Neszmélyi, Roland Pfister

**Affiliations:** ^1^ Department of Psychology 3, University of Würzburg, Germany; ^2^ Department of Psychology, Trier University, Germany; ^3^ Institute for Cognitive and Affective Neuroscience (ICAN), Trier University, Germany

**Keywords:** imitation, anticipation, expectation, group effects, action-effect integration

## Abstract

**Abstract:** Anticipating to be imitated by another agent primes corresponding action plans in action models. Here we assessed whether being imitated by more than one coactor boosts anticipated imitation. This prediction was based on corresponding findings from motor priming by perceiving rather than anticipating movements of multiple agents. In contrast to this previous work, the effects of anticipated imitation were similar for imitation by a single agent and joint imitation by two agents. Anticipated imitation, therefore, appears to be based on sparse representations of only selected features rather than including a full representation of all possible consequences of one’s own movements.


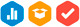


Imitation plays a critical role in everyday interactions, serving not only as a mechanism for skill acquisition (Byrne & Russon, 1998; Tomasello et al., 1993) but also as a catalyzer for a broad range of social processes (Meltzoff & Decety, 2003; Scassellati, 1999). There is substantial experimental evidence supporting the notion that behavior of the observer (imitator) is directly affected by the observed (model) actions: Actions executed by the model manifest spontaneously in the behavior of the observer (motor mimicry: Chartrand & Bargh, 1999), and imitators also initiate movements that are compatible with the model’s actions faster than incompatible ones even if the model’s actions are irrelevant with regard to the imitator’s task (i.e., automatic imitation: Brass et al., 2000; Heyes, 2011).

Interestingly, the model is also affected by imitation. This can manifest on the affective level (Dignath et al., 2018, 2021; van Baaren et al., 2004) but also in the planning and control of motor actions: Anticipated imitation (for a review, see Pfister et al., 2024) refers to the observation that reaction times of the model are faster when they are foreseeably imitated compared to them being counter-imitated. This is similar to the effects observed in response-effect compatibility studies, where actions come with shorter reaction times if they are followed by sensory effects that share some feature(s) with the actions (e.g., a left button-press followed by a sound coming from the left) compared to actions with incompatible effects (Kunde, 2001; Kunde et al., 2004; Pfister & Kunde, 2013). Anticipated imitation extends these findings by suggesting that social action effects (e.g., reactions of a coactor) might play a similar role in the planning, initiation, and control of actions as inanimate action effects (Kunde et al., 2018; Neszmélyi et al., 2022).

Imitation is usually thought of as a dyadic interaction with one model and one imitator. Recently, however, a few studies have also assessed automatic imitation in setups with more than one model (Cracco & Brass, 2018a, 2018b, 2018c; Cracco & Cooper, 2019; Cracco et al., 2015, 2016). The findings consistently show that compared to the single model scenario, the automatic imitation effect increases when the imitator’s action is primed by the synchronized action of multiple models. Current theories of automatic imitation usually presume that the effect is caused by the image of a hand movement activating motor patterns that induce that particular hand movement (for review, see Cracco et al., 2018; Heyes, 2011). Although the exact mechanisms are debated (e.g., associative links between action and effect: Cooper et al., 2013; common representations: Brass & Muhle-Karbe, 2014), these explanations suggest a link between the action and the visual effect associated with the movement (visual image of the moving hand). Due to these links, activating either the motor pattern responsible for the action or the representation of the action effect results in the automatic activation of the other. In the case of imitating multiple models, actions of multiple agents are represented concurrently by the observer’s motor system (Cracco et al., 2019; Cracco, Braem, & Brass, 2022; Cracco, Lee, et al., 2022). Each of the represented actions can support the initiation of the imitator’s own movement, and these facilitatory effects add up, resulting in a stronger automatic imitation effect (Cracco & Cooper, 2019; Cracco et al., 2016).

The predominant explanation of anticipated imitation proposes a very similar mechanism to the one described in the previous paragraph for automatic imitation (see Pfister et al., 2024): Both are based on bidirectional links between actions and the sensory effects of the actions. The main difference is that during anticipated imitation, motor patterns are not activated by perceiving the visual image of the hand movement but by anticipating, i.e., imagining, the hand movements that follow the action. It seems plausible to assume that increasing the number of expected imitator reactions would have a similar effect on anticipated imitation as increasing the number of perceived model actions has on automatic imitation. However, imitative settings with multiple imitators have received substantially less attention than settings with multiple models: Although situations with one model and multiple imitators are quite common in everyday interactions (e.g., a dance or sport instructor demonstrating a movement sequence to a group of students), currently there is only a single study that examined how the number of imitators affects the actions of the model. Galang et al. (2024) show in three experiments that the anticipated imitation effect, i.e., the reaction time difference between imitated and counter-imitated actions, is not influenced by the number of imitators.

However, several methodological points seem to limit the generalizability of the results reported by Galang et al. (2024). (1) Participants interacted with virtual imitators. (2) The imitators’ actions were presented in a first-person view, while anticipated imitation studies usually utilize a third person perspective. (3) The actions of the imitators were presented immediately after the actions of the model, while actual imitation involves a delay between the two agents’ actions. (4) The spatial aspect of the imitative task and the spatial arrangement of the two imitators might have interfered with each other. The possibility that these factors might have negatively influenced the results is supported by the fact that the anticipated imitation effect obtained by Galang et al. was small in comparison to those obtained in previous studies (i.e., ca. 4 ms).

In the current study, we examined anticipated imitation in an experimental setup where human participants were used for the manipulation of the number of imitators (instead of virtual coactors). This study can be regarded as complementary to the study of Galang et al. (2024) as it addresses the issues listed in the previous paragraph.^1^ On the other hand, limitations imposed on our experiment by the use of human participants as imitators (i.e., smaller sample size; timing of the imitators’ actions was not controlled) do not apply to the design of Galang et al. (2024). The main hypothesis of the two studies was also identical: We expected that the anticipated imitation effect would be more pronounced in a setting with two imitators as compared to the situation with a single imitator.

## Method

### Participants

A priori power analysis suggested that a sample of 34 participants can reveal an effect of medium magnitude^2^ (*d*_*z*_ = 0.5) with a power of .8 at an α level of .05. We collected data of 36 participants (*M*_age_ = 21.17, age range: 18–32, female: 31, male: 5, right-handed: 30, left-handed: 6, none of the participants met the predetermined exclusion criteria).

### Procedure

**Figure 1:**
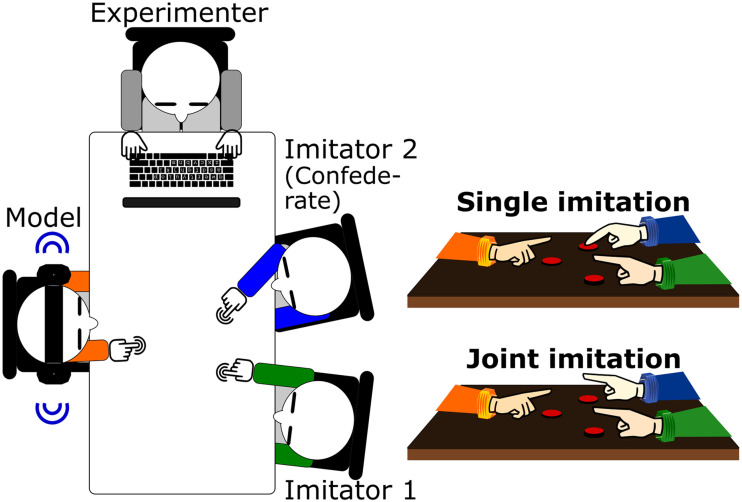
The experimental setup. The left panel displays how participants were seated during the experiment. The two panels on the right show the two experimental settings of single imitation and joint imitation.

Figure 1 shows the overall experimental setup. Two participants were invited to each session, and they were randomly assigned the roles of Model and Imitator 1 at the start of the experiment. A confederate participated as Imitator 2. The Model sat opposite the two imitators at a table. Each of them had a reaction time button in front of them that was fixed to the table. All agents were instructed to keep the button pressed down with their index finger, except when they were required to perform a finger lift, or during breaks between blocks. At the start of each trial, a syllable (*pa* or *ke*, spoken by a male voice) was presented to the Model via headphones. Depending on the syllable, the Model’s task was to release the button (raising the index finger visibly above it) for either a short (<400 ms) or a long time (between 600 and 1,000 ms). We manipulated the number of imitators and the imitator reaction independently. In the *single imitation setting*, only Imitator 1 reacted to the Model’s action (and Imitator 2 consistently kept the button pressed throughout the block), whereas in the *joint imitation setting*, both imitators were instructed to perform the same reaction. The imitator(s) were asked to perform the same action as the Model in the *imitation condition* and the opposite action in the *counter-imitation condition*. Participants completed two blocks of 36 trials for each combination of setting and condition. The setting and condition factors were both implemented in a blocked manner: That is, the setting (single vs. joint) was only changed at the halfway mark of the experiment, when participants had completed both conditions (imitation and counter-imitation). Within a given setting the condition was only changed when participants had completed both blocks of the condition. The order of the settings and of the conditions were randomly determined for each session. Before the experimental blocks, participants completed short practice blocks (12 trials) of each setting and condition. When all blocks with the initial role assignments had been finished, the two participants changed the roles of Model and Imitator 1. (The confederate remained in the role of Imitator 2 throughout the whole session.) After the change of roles, the whole experiment was repeated (with the same order of settings and conditions). Throughout the experiment, the Experimenter sat at the top of the table. The button presses of the three agents were displayed on the Experimenter’s screen who received notification if one of the agents made an error. In this case the task was interrupted, and the experimenter warned the participants about the error.

### Data Processing and Analysis

In the first block of each combination of setting and condition, the first 10 trials were excluded from all analyses, as were the first five trials of the second block. For the analysis of model errors, no further rejection criteria were applied. For the analysis of imitator errors, only trials were considered where the model and the confederate performed a correct action. All trials where the agent failed to perform the correct action were defined as error trials (i.e., wrong action, no action, action too early). Analyses were also performed with a narrower range of errors, considering only cases where participant performed the opposite action compared to the one that was indicated by the cue (i.e., short-long mix-up). Since the pattern revealed by the analyses did not differ meaningfully from the results reported for the wider error range, we only report the latter analyses. For the analysis of reaction times (RT), only actions were considered where the respective agent performed a correct action. Furthermore, imitator actions were only included in the RT analysis if the other two agents also performed the correct action. Trials were excluded from both model and imitator RT analyses, when an error was committed on the preceding trial by either of the three agents. Additionally, in both model and imitator RT analyses, we excluded trials with RTs 2.5 *SD* below or above the respective cell mean (per participant, setting, condition, and action type). The Model’s RT was calculated as the interval between the start of the sound playback, and the participant’s release of the reaction time button. The Imitator’s RT was measured as the interval between the Model’s pressing down of their button and the Imitator’s release of the button. Trial numbers retained for each analysis are reported in the Supplementary material.

We calculated the percentage of errors and the average RT for each participant, setting (single vs. joint), and condition (imitation vs. counter-imitation). These values were submitted to 2 × 2 repeated-measures analyses of variance (ANOVAs) with the factors Number of imitators (one vs. two) and Imitator reaction (imitation vs. counter-imitation). We also calculated the inclusion Bayes factor (Hinne et al., 2020) for all main effects and the interaction of the ANOVAs, by comparing all models with the term in question to all models without the term. Data were analyzed in *R* (version 4.3.1: R Core Team, 2023). R packages used for data analysis are listed in the Supplementary material. Further analyses (e.g., exploring the influence of confederate and action type) are also reported in the Supplementary material.

## Results

**Figure 2:**
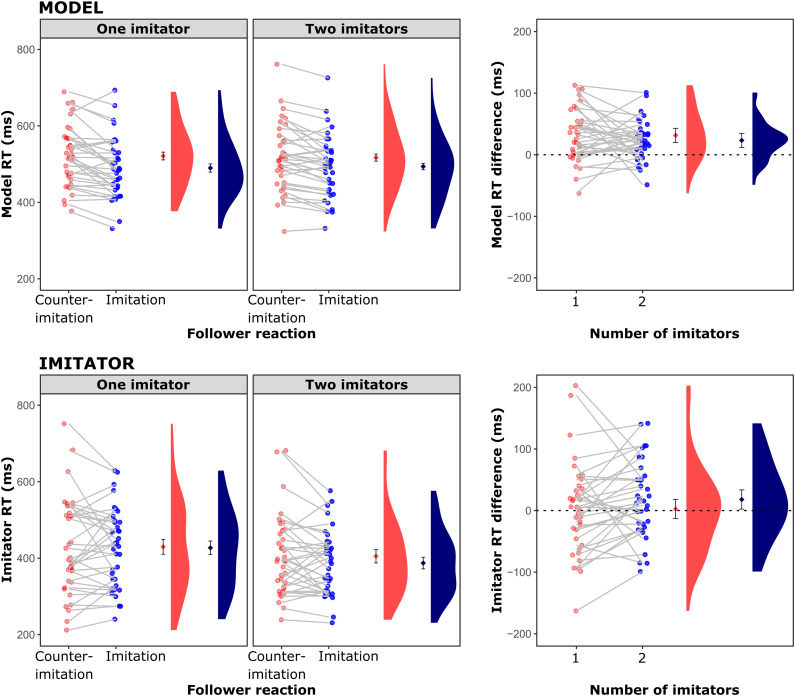
Reaction times of the Model (top row) and Imitator 1 (bottom row). Graphs on the left show reaction RTs separately for the two settings and conditions. Graphs on the right show the imitation effect—that is the counter-imitation minus imitation difference. A difference value of 0 (represented by dashed lines) shows that the condition (single imitation, joint imitation) had no effect. Values above zero indicate the conventional imitation effect, i.e., better performance in the imitation than in the counter-imitation condition. In each graph, single dots represent individual participants with gray lines connecting the data of a given participant. Next to the individual data points are group average values (with 95% within-subjects confidence intervals, Morey, 2008) and kernel density estimates for the distribution of individual values.

Figure 2 shows the results for the RT data across imitator and model responses. The ANOVA of the Model RTs indicated a significant Imitator reaction main effect, *F*(1, 35) = 32.61, *p* < .001, η_*p*_^2^ = .48, BF_inc_ > 10^5^. RTs were faster in the imitation condition (single imitation: *M* = 490 ms; joint imitation: *M* = 494 ms) compared to the counter-imitation condition (single imitation: *M* = 521 ms; joint imitation: *M* = 517 ms). Neither a main effect for Number of imitators, *F*(1, 35) = 0.05, *p* = .830, η_*p*_^2^ < .01, BF_inc_ = 0.18, nor an interaction were observed, however, *F*(1, 35) = 0.59, *p* = .447, η_*p*_^2^ = .02, BF_inc_ = 0.31.

The ANOVA of the imitator RTs indicated a significant main effect of Number of imitators, *F*(1, 35) = 11.35, *p* = .002, η_*p*_^2^ = .24, BF_inc_ = 74.05. Imitator reactions in the single imitation setting (imitation: *M* = 427 ms; counter-imitation: *M* = 430 ms) were initiated slower than in the joint imitation setting (imitation: *M* = 387 ms; counter-imitation: *M* = 405 ms). There was no main effect of Imitator reaction, *F*(1, 35) = 1.02, *p* = .319, η_*p*_^2^ = .03, BF_inc_ = 0.34, nor an interaction, *F*(1, 35) = 2.03, *p* = .163, η_*p*_^2^ = .05, BF_inc_ = 0.34.

The ANOVA of the model error rates did not reveal any significant effects, *p*s ≥ .287, BF_inc_ ≤ 0.32. The same was true for the ANOVA on the imitator’s error rates, *p*s ≥ .103, BF_inc_ ≤ 0.52. A more detailed description of the error rate analysis is presented in the online supplementary material (see Figure S1 in OSF).

## Discussion

In the present experiment, we replicated previous results on anticipated imitation (Lelonkiewicz et al., 2020; Pfister et al., 2013; Pfister et al., 2017; Weller et al., 2019, 2020): The Model’s RTs were significantly faster when their actions were followed predictably by imitative movements than when they were counter-imitated. Importantly, however, the results did not support our hypothesis that increasing the number of imitators would also increase the magnitude of the anticipated imitation effect. In fact, the Bayes factor provides moderate evidence for the null hypothesis, that is, it suggests that the anticipated imitation effect does not differ in the one- and two-imitator settings. This result resonates with the findings of Galang et al. (2024) who also found evidence against a positive connection between the number of imitators and the magnitude of the anticipated imitation effect. (Although their results were inconclusive with regard to the possibility of a reverse effect.) Taken together, the two studies provide strong support for the notion that increasing the number of imitators does not have the same effect on anticipated imitation as increasing the number of models does on automatic imitation. This is seemingly in contradiction with the idea that the two imitative effects supposedly rely on very similar mechanisms (Pfister et al., 2024). Both presuppose motor patterns responsible for a movement being activated by activating the sensory effects of the movement. The contradictory findings, however, might be explained by differences in the activation of the sensory effects.

Effect representations are activated in automatic imitation via perceiving a stimulus that is similar to the action effect, in anticipated imitation by anticipating/imagining the effect. Recent studies indicated that during action observation, multiple agents’ actions can be represented concurrently by the observer’s motor system (Cracco et al., 2019; Cracco, Braem, & Brass, 2022; Cracco, Lee, et al., 2022). To our knowledge, it has not been investigated yet whether this also applies to anticipating/imagining actions. A possibility could be that during effect anticipation, coordinated actions of the coactors (i.e., the imitators in the present case) are integrated into a single effect representation, due to distinctive features of the agents being less accessible compared to observation or because task-irrelevant effect features that could contribute to the representation of different agents are disregarded (Pfister et al., 2024). As a result, in the case of anticipation, increasing the number of agents that perform the same action would not have an influence on the initiation of the corresponding action. The idea that the role of the motor system is different in automatic and anticipated imitation and that distinctive features of biological movements play a more substantial role in the former effect might be supported by results showing that automatic imitation is sensitive to both spatial and anatomical effect features (Catmur & Heyes, 2011), whereas anticipated imitation solely relies on sparser representations that only include spatial features (Weller et al., 2019).

A difference between results of the current study and findings reported by Galang et al. (2024) is that the overall decrease of Model RTs with the increasing number of imitators observed in the online study was not found in the current experiment. The timing of the two imitators’ actions might provide an explanation for this inconsistency: In the study of Galang et al., the imitators’ actions were presented at the same time, immediately after the Model’s action. In the current study, the timing of the two imitators’ actions depended on their reaction time, and their actions were generally not perfectly synchronized. Moreover, the idea that the effect of imitator number requires perfect synchrony suggests that the effect is not related to social aspects of the task: A mechanism dedicated to the processing of social interactions would hardly work in real-life situations if it is not robust with regard to temporal variability.

Due to the use of human imitators instead of virtual ones, in addition to the Model’s actions, we could also assess the Imitator’s reactions. We found an overall decrease in Imitator RTs in the joint imitation setting. An obvious explanation for this could be that in this case, the participant in the Imitator 1 role imitated not only the model but also the second imitator (i.e., confederate), making the condition similar to an imitation task with multiple models (Cracco & Brass, 2018a, 2018b, 2018c; Cracco & Cooper, 2019; Cracco et al., 2015, 2016). Additional analyses, however, did not support this explanation (on trials where Imitator 2 reacted first, Imitator 1 RTs were not faster than their RTs on single imitation trials), and it is possible that the effect is caused by a competition between Imitators, or by Imitator 1’s anticipation of Imitator 2’s reaction (see Supplementary material 4.3). In apparent contradiction with previous studies (e.g., Brass et al., 2000; Weller et al., 2019, 2020), a significant RT difference between imitative and counter-imitative reactions was not observed for the imitator. A possible reason for this is that action initiation costs induced by model-imitator incompatibility might have been balanced out by gains resulting from compatibility of the two imitators’ actions.

In summary, our findings indicate that anticipated imitation remains relatively stable regardless of the number of imitators, in contrast to automatic imitation, where additional models strengthen the imitative response. This distinction may stem from the different ways in which perception versus anticipation or imagination activate action effect representations, shaping the planning and initiation of actions. Our results align with recent findings showing that action control mechanisms based on social action effect anticipation are remarkably similar to nonsocial processes (see Neszmélyi et al., 2022). Additionally, analysis of imitators’ RTs in joint imitation suggests that imitators are influenced not only by the model’s actions but also by those of fellow imitators. These insights open avenues for further investigation, potentially enriching our understanding of group interactions beyond the dyadic level.

## Footnotes

^1^
This complementary nature of the two studies is coincidental. The present experiment was conducted before publication of the Galang et al. (2024) study, without knowledge of that work.

^2^
In the current study, the effects of interest were assessed as interaction terms of 2 × 2 repeated-measures ANOVAs. Since there is no consensus on power analysis approaches for such effects, we based our power analysis on the fact that the interaction of a two-way repeated-measures ANOVA can also be assessed as a paired *t*-test on difference scores. Importantly, with this approach, the expected effect size is defined at the level of the difference variable rather than at the level of the original variable. This distinction complicates the interpretation of the relationship between the effect size used for power analysis and the effect sizes obtained in ANOVAs. Additionally, given that the sample size in the current study is lower than what more conservative estimations suggest (e.g., Brysbaert, 2019), the study might be underpowered to detect smaller effects.

## Notes

**Publication Ethics:** All participants signed informed consent. The experiment was in accordance with the Declaration of Helsinki, and it was approved by the ethics committee of the University of Würzburg.

**Authorship:** Bence Neszmélyi: Conceptualization, methodology, software, investigation, data curation, formal analysis, visualization, writing- original draft preparation. Roland Pfister: Conceptualization, methodology, resources, writing – reviewing and editing, supervision, funding acquisition.

**Open Science:** To the best of our ability and knowledge, we have provided all original materials and clear references to all other materials via a stable online repository.

Open Data: Data and analysis scripts needed to reproduce all of the reported results are available at https://osf.io/bc8p7/ (Neszmélyi & Pfister, 2025).

Open Materials: Information and software needed to reproduce all of the reported methodology is available at https://osf.io/bc8p7/ (Neszmélyi & Pfister, 2025).

Preregistration and Analysis Plan: This study was preregistered at https://aspredicted.org/yz8w-2xtc.pdf.

The online supplementary materials are available at https://osf.io/bc8p7/ (Neszmélyi & Pfister, 2025).
